# Consenso latinoamericano para definir, categorizar y notificar patógenos multirresistentes, con resistencia extendida o panresistentes

**DOI:** 10.26633/RPSP.2019.65

**Published:** 2019-08-22

**Authors:** María Antonieta Jiménez Pearson, Marcelo Galas, Alejandra Corso, Juan C. Hormazábal, Carolina Duarte Valderrama, Nuris Salgado Marcano, Pilar Ramón-Pardo, Roberto G. Melano

**Affiliations:** 1 Instituto Costarricense de Investigación y Enseñanza en Nutrición y Salud (INCIENSA), Tres Ríos Instituto Costarricense de Investigación y Enseñanza en Nutrición y Salud (INCIENSA), Tres Ríos Cartago Costa Rica Instituto Costarricense de Investigación y Enseñanza en Nutrición y Salud (INCIENSA), Tres Ríos, Cartago, Costa Rica.; 2 Organización Panamericana de la Salud Organización Panamericana de la Salud Washington, DC Estados Unidos de América Organización Panamericana de la Salud, Washington, DC, Estados Unidos de América.; 3 Servicio Antimicrobianos, Instituto Nacional de Enfermedades Infecciosas-Administración Nacional de Laboratorios e Institutos de Salud (INEI-ANLIS) “Dr. Carlos G. Malbrán” Servicio Antimicrobianos, Instituto Nacional de Enfermedades Infecciosas-Administración Nacional de Laboratorios e Institutos de Salud (INEI-ANLIS) “Dr. Carlos G. Malbrán” Buenos Aires Argentina Servicio Antimicrobianos, Instituto Nacional de Enfermedades Infecciosas-Administración Nacional de Laboratorios e Institutos de Salud (INEI-ANLIS) “Dr. Carlos G. Malbrán”, Buenos Aires, Argentina.; 4 Departamento de Laboratorio Biomédico Departamento de Laboratorio Biomédico Instituto de Salud Pública de Chile Santiago Chile Departamento de Laboratorio Biomédico, Instituto de Salud Pública de Chile, Santiago, Chile.; 5 Grupo de Microbiología Grupo de Microbiología Instituto Nacional de Salud Bogotá Colombia Grupo de Microbiología, Instituto Nacional de Salud, Bogotá, Colombia.; 6 Sección de Aislamiento e Identificación bacteriana Sección de Aislamiento e Identificación bacteriana Instituto Nacional de Higiene “Rafael Rangel” Caracas Venezuela Sección de Aislamiento e Identificación bacteriana, Instituto Nacional de Higiene “Rafael Rangel”, Caracas, Venezuela.; 7 Laboratorio de Salud Pública de Ontario Laboratorio de Salud Pública de Ontario Toronto, Ontario Canadá Laboratorio de Salud Pública de Ontario, Toronto, Ontario, Canadá.; 8 Los autores de la ReLAVRA y sus afiliaciones se mencionan al final del manuscrito. Los autores de la ReLAVRA y sus afiliaciones se mencionan al final del manuscrito. Los autores de la ReLAVRA y sus afiliaciones se mencionan al final del manuscrito.

**Keywords:** Antimicrobianos, farmacorresistencia bacteriana, bacterias gramnegativas, consenso, América Latina, Anti-infective agents, drug resistance, microbial, Gram-negative bacteria, consensus, Latin America, Anti-infecciosos, resistência microbiana a medicamentos, bactérias gram-negativas, consenso, América Latina

## Abstract

Se presenta un consenso latinoamericano que permite estandarizar las definiciones de los diferentes niveles de resistencia a los antimicrobianos en bacterias de importancia en salud pública. Se describen los criterios de inclusión y exclusión para las metodologías a utilizar y para los antibióticos a incluir (por disponibilidad, relevancia y existencia de puntos de corte). Como propuesta piloto se eligieron tres microorganismos gramnegativos de gran impacto en el ambiente hospitalario (*Klebsiella pneumoniae*, *Pseudomonas aeruginosa* y *Acinetobacter* spp.). La falta de puntos de corte para ciertos antibióticos (por ejemplo, tigeciclina, fosfomicina y colistina), claves para el tratamiento de infecciones causadas por estos patógenos que presentan multirresistencia o resistencia extendida, llevó a la necesidad de discutir y consensuar puntos de corte provisorios para la vigilancia de la resistencia a estos fármacos. Se abordó y consensuó también el uso de pruebas de sensibilidad alternativas a los métodos aprobados por las guías internacionales, de aplicación más sencilla como pruebas de rutina en los laboratorios de bacteriología clínica. El principal beneficio de este documento es proporcionar a los laboratorios latinoamericanos un marco estandarizado y consensuado para la identificación y la vigilancia constante y unificada de microorganismos resistentes. Las recomendaciones incluidas en este documento son el resultado consensuado por los representantes de los laboratorios nacionales de referencia de los países que integran la Red Latinoamericana de Vigilancia de la Resistencia a los Antibióticos coordinada por la Organización Panamericana de la Salud.

## Abstract (en)

This document presents a Latin American consensus to standardize definitions of different levels of antimicrobial resistance in bacteria of public health importance. Inclusion and exclusion criteria are described for antibiotics to include (availability, relevance, and existence of cut-off values) and for methodologies to use. Three gram-negative microorganisms with a great impact in the hospital environment (*Klebsiella pneumoniae*, *Pseudomonas aeruginosa,* and *Acinetobacter* spp.) were selected as a pilot proposal. The lack of cut-off values for certain antibiotics (e.g., tigecycline, fosfomycin, and colistin), crucial in treating infections caused by multi-drug resistant or extensively drug-resistant pathogens, led to the need to discuss and agree on provisional cut-off values for monitoring resistance to these drugs. The work team also addressed and reached consensus on easier-to-use alternative susceptibility tests, other than methods approved by international guidelines, for routine testing in clinical bacteriology laboratories. The main benefit of this document is to provide Latin American laboratories with a standardized and consensual framework for the identification and constant and unified surveillance of resistant microorganisms. The recommendations included in this document are the result of consensus among representatives of the national reference laboratories in the countries belonging to the Latin American Surveillance Network of Antimicrobial Resistance, coordinated by the Pan American Health Organization.

## RESUMO

É apresentado um consenso latino-americano para padronizar a definição dos graus de resistência antimicrobiana em bactérias de importância em saúde pública. São descritos os critérios de inclusão e exclusão para os antibióticos a serem incluídos (disponibilidade, relevância e pontos de corte de sensibilidade) e metodologias a serem usadas. Como proposta-piloto, foram selecionados três microrganismos Gram-negativos de grande impacto no ambiente hospitalar (*Klebsiella pneumoniae*, *Pseudomonas aeruginosa* e *Acinetobacter* spp.). Diante da falta de pontos de corte para alguns antibióticos (como tigeciclina, fosfomicina e colistina), essenciais para o tratamento de infecções causadas por patógenos com multirresistência ou resistência ampliada, foram debatidos e aprovados pela maioria pontos de corte provisórios para a vigilância da resistência a estes fármacos. Também foi discutido e aprovado o uso de testes de suscetibilidade alternativos aos métodos aprovados pelas diretrizes internacionais, mais simples de serem realizados como testes de rotina nos laboratórios de bacteriologia clínica. A principal contribuição deste documento é oferecer aos laboratórios latino-americanos um sistema padronizado e consensual para a identificação de microrganismos resistentes e a vigilância contínua e uniforme destes patógenos. As recomendações aqui contidas foram feitas por consenso por representantes dos laboratórios nacionais de referência dos países que integram a Rede Latino-Americana de Vigilância da Resistência Antimicrobiana, coordenada pela Organização Pan-Americana da Saúde (OPAS).

El uso de antibióticos, desde sus inicios en la primera mitad del siglo XX, se constituyó en una de las piedras angulares de la medicina moderna y permitió salvar millones de vidas. Sin embargo, su efectividad se vio disminuida por la emergencia de patógenos resistentes a estos medicamentos, una consecuencia inevitable regida por la selección natural descrita por Charles Darwin (1). La presión selectiva de los antimicrobianos, producida en su mayoría por hongos y bacterias de origen ambiental, llevó a una coevolución lógica: los microorganismos productores, o aquellos que comparten su nicho ecológico, desarrollaron mecanismos de resistencia a esos mismos compuestos como parte de su propia subsistencia (2). De esta forma, la presencia de antibióticos en un ambiente determinado no solo selecciona cambios en genes propios de especies que buscan sobrevivir a su acción, sino que también favorece la diseminación lateral de estos mecanismos desde sus huéspedes originales a otras especies bacterianas a través de elementos genéticos móviles como transposones y plásmidos. En el ambiente hospitalario, la presión antibiótica selectiva es enorme por el uso de antimicrobianos de amplio espectro, y por ello no sorprende la emergencia de patógenos multirresistentes (3). Además, la mayor necesidad de alimentos y la cría intensiva de animales para consumo humano ha propiciado la utilización de estos fármacos en veterinaria y también en la industria de producción de alimentos (4, 5). La utilización de antibióticos en estas áreas dificulta aún más el control de su uso racional y genera una nueva fuente de selección de bacterias resistentes que luego pueden diseminarse mediante los alimentos o el medio ambiente, como así también colonizar el tracto digestivo de animales, incluidos los seres humanos. Como consecuencia, nos dirigimos hacia una era similar a la preantibiótica, con infecciones bacterianas para las cuales no hay tratamiento disponible (6), o en la cual las alternativas están lejos de ser ideales (por ejemplo, antibióticos antiguos en desuso por su elevada toxicidad, como las polimixinas). Sin ser aún la regla, estos casos son cada vez más frecuentes. En vista del escaso número de antibióticos nuevos en desarrollo (7), se vuelve fundamental la detección y monitorización de bacterias multirresistentes para así definir acciones que mejoren el uso de los antimicrobianos disponibles y prevenir la diseminación de patógenos resistentes.

Un paso necesario en este proceso es establecer un consenso en la definición de las diferentes categorías de resistencia que un microorganismo puede expresar. Se han descrito tres categorías generales, denominadas multirresistencia (MDR, del inglés *multidrug-resistance*), resistencia extendida (XDR, del inglés *extensively drug-resistance*) y panresistencia (PDR, del inglés *pandrug-resistance*), sin un acuerdo en cuanto a sus definiciones (8, 9). Los primeros intentos colaborativos en este sentido se plasmaron en dos publicaciones: una iniciativa conjunta de los Centros para la Prevención y Control de Enfermedades de la Unión Europea y de los Estados Unidos de América (10), y una iniciativa de la Red Canadiense de Laboratorios de Salud Pública (11, 12). En ambos consensos, el objetivo fue proveer definiciones normalizadas acerca de las formas más apropiadas de detectar y reportar un fenotipo adquirido de resistencia múltiple a antibióticos. En ellos se ponen de manifiesto una serie de criterios de inclusión y exclusión para encuadrar estas definiciones en un marco estándar, y se especifican los listados de antibióticos a probar para cada uno de los microorganismos considerados. Ambos estudios excluyen los mecanismos de resistencia intrínsecos, los cuales se consideran dentro del fenotipo salvaje de esas especies bacterianas. Sin embargo, los criterios utilizados para esas definiciones no se ajustan a las necesidades de América Latina, ya sea por el tipo de antibióticos incluidos, por las diferentes capacidades de los países involucrados o por la epidemiología de la resistencia local.

Por esas razones, el objetivo de este artículo es crear criterios estándares para definir los fenotipos de resistencia con base en una lista de antibióticos comunes y en el uso de las mismas metodologías y puntos de corte, establecidos por consenso entre los países de América Latina. Se propone una terminología regional estandarizada que permita describir perfiles de resistencia adquiridos de importancia en salud pública y promover su notificación y monitorización.

Las recomendaciones incluidas en este trabajo son el resultado de las discusiones y el consenso logrado por los representantes de los laboratorios nacionales de referencia de los países que integran la Red Latinoamericana de Vigilancia de la Resistencia a los Antibióticos (ReLAVRA) coordinada por la Organización Panamericana de la Salud/Organización Mundial de la Salud (OPS/OMS). La ReLAVRA fue creada en 1996 con el apoyo de la OPS con el fin de obtener datos microbiológicos fidedignos, oportunos y reproducibles para mejorar la atención del paciente y fortalecer la vigilancia de la resistencia a los antimicrobianos mediante programas de garantía de calidad sostenibles. Se elaboraron estas recomendaciones para ser utilizadas por todos los laboratorios latinoamericanos de microbiología clínica humana, a fin de estandarizar la nomenclatura de categorización de los niveles de resistencia en bacterias patógenas causantes de infecciones comunitarias o asociadas a la atención de la salud.

Este documento resume el trabajo iniciado en la reunión bienal conjunta de ReLAVRA y la Red Interamericana de Laboratorios de Análisis de Alimentos en Montevideo, Uruguay, el 30 de noviembre de 2017. Representantes de todos los países que en la actualidad forman parte de ReLAVRA participaron de la elaboración de este documento: Argentina, Belice, Bolivia, Brasil, Chile, Colombia, Costa Rica, Cuba, Ecuador, El Salvador, Guatemala, Honduras, México, Nicaragua, Panamá, Paraguay, Perú, República Dominicana, Uruguay y Venezuela. Se elaboró un informe inicial con una consulta general acerca de los criterios a emplear y los antibióticos a incluir, seguida de una encuesta en línea en la que se votaron los puntos en discusión. Con estos datos se escribió el primer borrador, el cual fue revisado por representantes de cuatro centros nacionales de referencia (determinados en la reunión de Montevideo). Como último paso, el consenso final revisado fue distribuido a todos los centros nacionales de referencia que conforman la ReLAVRA para su aprobación.

## Criterios de inclusión y de exclusión

Para la elaboración de este consenso se emplearon como base las publicaciones antes mencionadas (10-12). Además, se tomó en cuenta un documento de la OMS donde se enlistan las bacterias prioritarias resistentes a los antibióticos, en la que se incluyen las 12 familias de bacterias patógenas multirresistentes de mayor riesgo para la salud humana, encabezadas por *Acinetobacter baumannii*, *Pseudomonas aeruginosa* y *Enterobacteriaceae* resistentes a antibióticos carbapenémicos (13).

Como propuesta piloto se eligieron tres microorganismos gramnegativos de gran impacto en el ambiente hospitalario: *Klebsiella pneumoniae*, *P. aeruginosa* y *Acinetobacter* spp. (cuadro 1). En actualizaciones futuras se incluirán otras bacterias de importancia clínica en la Región. Dada la permanente evolución y emergencia de nuevos mecanismos de resistencia a los antimicrobianos, los cambios de los puntos de corte de los antibióticos para las pruebas de sensibilidad incluidas en las guías internacionales como así también de los métodos de sensibilidad, y la introducción y uso de nuevos antimicrobianos aprobados en los diferentes países de la Regións; la actualización de las definiciones y de este documento en general se consensuarán cada dos años, durante las reuniones de ReLAVRA, o mediante reuniones virtuales en línea si así se requiriese.

**CUADRO 1.** Reglas para la definición de multirresistencia, resistencia extendida y panresistencia en aislamientos clínicos de *Klebsiella pneumoniae, Pseudomonas aeruginosa y Acinetobacter* spp.

Definición	Grupos de antibióticos
Klebsiella pneumoniae	
	Amoxicilina-ácido clavulánico o ampicilina-sulbactam
	Piperacilina, tazobactam
	Ceftazidima o cefotaxima/ceftriazona o cefepima
	Imipenem o meropenem
MDR: resistente a 3 de los 12 grupos de antibióticos	Aztreonam
XDR: resistente a 10 u 11 de los 12 grupos de antibióticos	Gentamicina
PDR: resistente a todos los grupos de antibióticos	Amikacina
	Ciprofloxacino
	Trimetoprima-sulfametoxazol
	Fosfomicina
	Tigeciclina
	Colistina
Pseudomonas aeruginosa	
	Piperacilina, tazobactam
	Ceftazidima
	Cefepima
MDR: resistente a 3 de los 10 grupos de antibióticos	Aztreonam
XDR: resistente a 8 o 9 de los 10 grupos de antibióticos	Imipenem
PDR: resistente a todos los grupos de antibióticos	Meropenem
	Gentamicina
	Amikacina
	Ciprofloxacino o levofloxacino
	Colistina
*Acinetobacter* spp.	
	Ampicilina-sulbactam
	Piperacilina, tazobactam
	Ceftazidima o cefepima
MDR: resistente a 3 de los 11 grupos de antibióticos	Imipenem o meropenem
XDR: resistente a 9 o 10 de los 11 grupos de antibióticos	Gentamicina
PDR: resistente a todos los grupos de antibióticos	Amikacina
	Ciprofloxacino
	Trimetoprima-sulfametoxazol
	Minociclina
	Tigeciclina
	Colistina

Los criterios de inclusión y de exclusión empleados fueron: 1) los antibióticos a ser usados (su disponibilidad en los países de la Región y relevancia para el tratamiento, y los puntos de corte para cada uno de ellos), y 2) las pruebas de sensibilidad recomendadas (aprobadas por las guías internacionales vigentes).

## Lista de antibióticos

Se consensuó tener en cuenta solo los grupos de antibióticos que comúnmente se prueban en los laboratorios clínicos de la Región para la monitorización de los fenotipos de resistencia y que, además, son relevantes para el tratamiento de infecciones causadas por el patógeno en cuestión. Sin embargo, esta lista debería adecuarse a los aprobados en cada país, de acuerdo al vademécum local. Por ejemplo, los países que disponen de antimicrobianos nuevos para el tratamiento de infecciones bacterianas deberían incluirlos en la lista de antibióticos a ser probados por los laboratorios locales. De igual forma, un antibiótico incluido en este documento no debería probarse si no está disponible para su uso clínico en el país.

La separación de algunos antibióticos en grupos diferentes, aunque pertenezcan a la misma familia, se basó en el hecho de que ciertos mecanismos de resistencia son exclusivos o pueden presentarse como disociaciones (p. ej., un aislamiento clínico podría ser resistente a ceftazidima, pero sensible [S] a cefepima; lo mismo sucede con imipenem y meropenem en el caso de *P. aeruginosa*) (cuadro 1).

Cada antibiótico incluido debe tener puntos de corte definidos por alguna de las siguientes guías internacionales vigentes: el Instituto de Normas Clínicas y de Laboratorio (CLSI, por sus siglas en inglés) (14), el Comité Europeo sobre Pruebas de Susceptibilidad Antimicrobiana (EUCAST, por sus siglas en inglés) (15), Administración de Alimentos y Medicamentos de los Estados Unidos de América (FDA, por sus siglas en inglés) (16), o los definidos por el Laboratorio de Referencia Regional (LRR, Servicio Antimicrobianos del Instituto Nacional de Enfermedades Infecciosas-ANLIS “Dr. C. G. Malbrán”, Argentina) y consensuados con los países miembros de ReLAVRA.

La tigeciclina es una de las pocas opciones terapéuticas para el tratamiento de infecciones debidas a patógenos bacterianos con resistencia múltiple (p. ej., *K. pneumoniae* y *Acinetobactr* spp.), y el monitoreo de la resistencia a este fármaco es fundamental desde el punto de vista clínico y epidemiológico. Por este motivo, la tigeciclina será la única excepción al criterio “cada antibiótico incluido en este documento debe tener puntos de corte definidos” (véase más adelante). Si bien se utilizaron los puntos de corte del CLSI para la mayoría de los antibióticos incluidos, estas guías no poseen puntos de corte para fosfomicina y colistina para enterobacterias. Por ello, se consensuaron puntos de corte para estos tres antibióticos.

## Pruebas de sensibilidad

Se utilizarán los métodos aprobados por el CLSI, el EUCAST y la FDA: difusión por discos (DD), microdilución y macrodilución en caldo o dilución en agar, con base en las pautas del CLSI para las pruebas en Enterobacteriales, *P. aeruginosa* y *Acinetobacter* spp., así como también los métodos automatizados o las tiras con gradientes de antibióticos aprobados.

En el caso de la colistina, un grupo de trabajo conjunto del CLSI y EUCAST recomendó la microdilución en caldo como único método para evaluar la sensibilidad a este fármaco en Enterobacteriales, *P. aeruginosa* y *Acinetobacter* spp., debido a las tasas de errores altas e inaceptables de los otros métodos (17). Sin embargo, esta técnica es laboriosa y no se realiza de manera sistemática en los laboratorios clínicos. Por ese motivo, el LRR evaluó algunos métodos alternativos que podrían usarse en los laboratorios clínicos a fin de contar con una técnica práctica y precisa para la detección de aislamientos resistentes a colistina hasta que se aprueben nuevas normativas por los entes internacionales mencionados.

## Definiciones propuestas

Las definiciones de fenotipo de resistencia y la lista de antibióticos que se presentan en este trabajo son para uso en salud pública y con fines epidemiológicos. No pretenden reemplazar la lista de antibióticos utilizados en los laboratorios para elaborar reportes clínicos, contribuir a la toma de decisiones terapéuticas u ofrecer una guía en las prácticas de control de infecciones, sino facilitar la detección, la notificación y la monitorización de patógenos con resistencia múltiple. Los listados finales de antibióticos consensuados por los representantes de ReLAVRA para cada patógeno incluido en este trabajo se detallan en el cuadro 1.

Se consensuó utilizar la definición de la categoría ‘Resistente’ (R) en lugar de ‘no sensible’ (categoría ‘Intermedia’ [I] más R) para evitar que la definición de MDR, XDR y PDR por los laboratorios clínicos sea confusa debido al análisis adicional de los datos de “resistencia intermedia”. Esto significa que se incluirán para su categorización solo los antibióticos a los que, según el punto de corte, los patógenos analizados sean resistentes.

Se determinaron las siguientes definiciones de fenotipo de resistencia, comunes a los tres patógenos incluidos en este documento:

MDR: el aislamiento bacteriano es resistente al menos a tres de los grupos de antibióticos incluidos en el cuadro 1.

XDR: el aislamiento bacteriano es resistente a todos los grupos de antibióticos excepto a uno o dos de ellos; es decir, se mantiene S o I solo a uno o dos grupos de antibióticos incluidos en el cuadro 1.

PDR: el aislamiento bacteriano es resistente a todos los antibióticos incluidos en el cuadro 1.

Queda establecido que, para que un aislamiento pueda ser definido como PDR, todos los antibióticos de la lista (adaptada según el vademécum local) deben haber sido probados con métodos de referencia; de lo contrario, será definido como MDR, XDR y como potencial PDR hasta que se prueben todos los antibióticos de la lista o se evalúe con la metodología adecuada, ya sea por un laboratorio clínico o por el laboratorio de referencia nacional o regional.

## Puntos de corte para tigeciclina, fosfomicina y colistina

Se consensuaron los siguientes puntos de corte:

- Tigeciclina: en las guías EUCAST de 2018, los puntos de corte para concentración inhibitoria mínima (CIM) para enterobacterias eran R ≥ 4 µg/mL, S ≤1 µg/mL. Se consensuó utilizarlos en el caso de *K. pneumoniae* y extender su uso a *Acinetobacter* spp. Sin embargo, en 2019 EUCAST no solo cambió esos puntos de corte (los redujo a R ≥ 1 µg/mL, S ≤ 0,5 µg/mL) sino que además solo se aplican a *Escherichia coli* y *Citrobacter koseri*. De esa manera, tanto EUCAST como CLSI no disponen de puntos de corte para Enterobacteriales ni *Acinetobacter* spp., mientras que los definidos por FDA para *Enterobacteriaceae* son muy elevados (R ≥ 8 µg/mL, S ≤ 2 µg/mL) si se tiene en cuenta la farmacocinética y la farmacodinamia de este antibiótico. Por estas razones, tanto para *K. pneumoniae* como para *Acinetobacter* spp. se consensuó utilizar los puntos de corte para CIM de EUCAST 2018 para Enterobacteriales (R ≥ 4 µg/mL, I = 2 µg/mL, S ≤ 1 µg/mL) hasta que se actualicen nuevos puntos de corte para estos patógenos. Existe una recomendación de EUCAST que incluye bibliografía que apoya esta decisión (18). Para DD, se consensuó utilizar, para ambos microorganismos, los puntos de corte definidos por el LRR (R ≤ 16 mm, I = 17-20 mm, S ≥ 21 mm), los cuales son diferentes a los de EUCAST (que solo aplican a *E. coli*). Estos puntos de corte del LRR se basaron en un estudio que incluyó una mayoría de aislamientos clínicos de *K. pneumoniae* (N = 195 enterobacterias: 158 *K. pneumoniae*, 24 *Enterobacter* spp., 6 *Serratia marcescens*, 3 *Citrobacter freundii*, 2 *Klebsiella oxytoca*, 1 *E. coli*, y 1 *Providencia rettgeri*) (19), por lo que se ajustan mejor a la distribución de halos de inhibición para tigeciclina en esta especie bacteriana. Estos puntos de corte se aplicarán en forma provisoria solo para definir el estatus de resistencia de estas especies bacterianas y no deben usarse como un criterio clínico para reportar al aislamiento. Además, su uso se modificará cuando alguna de las guías internacionales actualice los puntos de corte para este antibiótico en estos patógenos. Cabe aclarar que todo aislamiento con sensibilidad intermedia mediante el método de difusión por discos debe ser confirmado con la determinación de la CIM para este antibiótico.
- Fosfomicina: en el caso de *K. pneumoniae* se consensuó utilizar los puntos de corte de CIM para fosfomicina intravenosa de EUCAST para enterobacterias (R ≥ 64 µg/mL, S ≤ 32 µg/mL) (23), y el del LRR para DD (R ≤ 15 mm, I = 16 mm, S ≥ 17 mm, con discos que contengan 200 μg de fosfomicina más 50 μg de glucosa-6-fosfato, y al igual que las recomendaciones de EUCAST, no deben considerarse las colonias dentro del halo de inhibición) (19). Este último criterio se consensuó por los siguientes motivos: 1) muchos de los laboratorios clínicos de la región usan la DD como método de tamizaje sistemático, 2) se han descrito genes homólogos a *fosA* (la enzima FosA confiere resistencia a la fosfomicina mediante la conjugación de glutatión con el antibiótico) en los genomas de *Klebsiella* spp., *S. marcescens* y *Enterobacter* spp., lo que explicaría el nivel de resistencia incrementado a fosfomicina en estos géneros (20, 21); (3) los puntos de corte de CLSI y EUCAST para DD se definieron solo para *E. coli*, que no posee un mecanismo intrínseco de resistencia a fosfomicina, y por lo dicho en el segundo punto no pueden ser adaptados a *K. pneumoniae*; y 4) los puntos de corte definidos por el LRR se basaron en el mismo estudio mencionado para tigeciclina (para fosfomicina se estudiaron 181 de los 195 aislamientos, con una mayoría de *K. pneumoniae*) por lo que se ajustan mejor a la distribución de halos de inhibición para fosfomicina en esta especie bacteriana (19).
- Colistina: en el caso de *K. pneumoniae* se consensuó usar los puntos de corte de EUCAST para Enterobacteriales (R ≥ 4 µg/mL, S ≤ 2 µg/mL) (15). Para *P. aeruginosa* y *Acinetobacter* spp., se consensuaron los del CLSI/EUCAST (R ≥ 4 µg/mL, S ≤ 2 µg/mL) (14, 15).

## Pruebas de sensibilidad recomendadas para colistina

Se consensuó el uso de los siguientes métodos:

- Métodos de referencia: microdilución y macrodilución en caldo (los métodos de referencia primarios), y dilución en agar (si bien no es un método recomendado por CLSI/EUCAST, varios estudios han demostrado una fuerte correlación con la dilución en caldo) (22-24).
- Métodos alternativos: Sensititre^®^ (Thermo Fisher Scientific, MA, EE.UU.) (se encontró 96-98% de concordancia [CA, por las siglas en inglés de *categorical* *agreement*] con la microdilución en caldo y 0% de falsa sensibilidad) (24, 25), el sistema automatizado MicroScan® (Beckman Coulter, CA, EE.UU.) (CA de 87% para *Acinetobacter* spp. y una sensibilidad de 88% para la detección de resistencia a polimixina B en aislamientos de *K. pneumoniae*; los resultados de no-sensibilidad en bacterias no fermentadoras deberían ser confirmados por un método de referencia) (26-28), predifusión con tabletas de colistina (CA 97-100%), colistina Agar Spot (CA 99,5-100%) y elución de discos de colistina (CA 98-100%) (29).

Algunos métodos presentan discrepancias de importancia con los métodos de referencia, las cuales se hallan asociadas sobre todo a falsa sensibilidad. Se incluyen la DD (30), tiras de gradiente (24, 31), y los sistemas automatizados Vitek®2 (bioMérieux, Marcyl’Étoile, Francia) (22, 31) y Phoenix^®^ (Becton Dickinson Diagnostics, Sparks, MD, EE.UU.) (28, 32). No se recomiendan estos métodos para determinar el fenotipo de resistencia a la colistina.

Para mayores detalles acerca de las limitaciones de ciertas metodologías para establecer el perfil de sensibilidad a colistina, se puede consultar en publicaciones recientes (32-34) y en el “Boletín Informativo No. 30: Desafíos en los métodos de evaluación de la sensibilidad a polimixinas (colistina/polimixina B), setiembre 2017”, del Programa Latinoamericano de Control de Calidad en Bacteriología y Resistencia a los Antimicrobianos, diseñado por el LRR (35).

## Reporte a los laboratorios nacionales de referencia

Los laboratorios clínicos deben notificar y remitir a los laboratorios nacionales de referencia todo aislamiento PDR como así también los XDR sospechosos de ser PDR. El aislamiento debe ser remitido junto a la información epidemiológica del paciente (edad, sexo, historia de viajes fuera del país en los últimos tres meses, diagnóstico, infección asociada a la atención de la salud o adquirida en la comunidad), las características de la muestra (tipo de muestra de la que se recuperó el aislamiento bacteriano y fecha del aislamiento) y el perfil de sensibilidad obtenido por el laboratorio remitente. Si se tiene más de un aislamiento de la misma especie y perfil de sensibilidad recuperados del mismo paciente, se recomienda enviar solo el primer aislamiento PDR o potencialmente PDR.

Los laboratorios nacionales de referencia han de ser capaces de definir un fenotipo PDR y deben promover el desarrollo de capacidad en los laboratorios clínicos, para que estos definan qué tipo de resistencia fenotípica tiene un microorganismo. Además, se acordó que los laboratorios nacionales de referencia con mayores capacidades apoyarían a los que así lo requieran, a través de la OPS. Cada laboratorio de referencia nacional centralizará la información epidemiológica acerca de los aislamientos PDR de su respectivo país, la cual se reportará a entes gubernamentales (p. ej., el ministerio de salud) a fin de implementar políticas para limitar la diseminación a nivel local, y a la OPS para el análisis del riesgo, el apoyo para el control y la comunicación a los otros países de la Región.

En resumen, la contención de la resistencia a los antimicrobianos comienza en su detección y caracterización en el laboratorio de microbiología. Por lo tanto, es de importancia fundamental disponer de un documento de consenso que unifique las prácticas de los laboratorios de América Latina y permita una robusta vigilancia a nivel local, nacional y regional sobre la cual basar de manera oportuna las medidas adecuadas para la prevención de la diseminación de estos patógenos para los que no existe tratamiento antibiótico eficaz. La falta de puntos de corte para ciertos antibióticos clave para el tratamiento de infecciones causadas por las bacterias incluidas en este trabajo, fármacos de último recurso cuando estos microorganismos son MDR o XDR, llevó a la necesidad de discutir y consensuar alternativas regionales para obtener resultados relacionados a estos antibióticos. De igual manera, se abordó y consensuó el uso de métodos alternativos, de implementación más sencilla que los aprobados, en los laboratorios de bacteriología clínica. En ambos casos, las conclusiones consensuadas son provisorias y estarán vigentes hasta que alguna de las guías internacionales consideradas publique nuevas normativas referentes a estos puntos. Así, este documento proporciona a los laboratorios latinoamericanos un marco estandarizado y consensuado para la identificación y la vigilancia constante y unificada de microorganismos resistentes, que son el reflejo de los antimicrobianos empleados en el continente.

## Contribución de los autores.

MAJP, MG, PRP y RGM concibieron el estudio original y redactaron el artículo en forma conjunta. Todos los autores discutieron y consensuaron los datos presentados en este estudio. MAJP, MG y RGM recolectaron y analizaron los datos. MAJP, MG, AC, JCH, CDV, NSM, PRP y RGM contribuyeron con la revisión del texto. Todos los autores revisaron y aprobaron la versión final.

## Miembros de la ReLAVRA.

Los participantes de los laboratorios nacionales de referencia que integran la Red Latinoamericana de Vigilancia de la Resistencia a los Antimicrobianos, coautores de este trabajo, son: C. Lucero, Instituto Nacional de Enfermedades Infecciosas “Dr. C. Malbrán” Buenos Aires, Argentina; A. Sosa, Central Medical Laboratory, Belice; E. Damiani, Instituto Nacional de Laboratorios de Salud, La Paz, Bolivia; S. Seara Cordeiro, Coordenação Geral de Laboratórios, Brasilia, Brasil; S. Prat, Instituto de Salud Pública, Santiago, Chile; M. V. Ovalle, Instituto Nacional de Salud, Bogotá, Colombia; H. Bolaños, Instituto Costarricense de Investigación y Enseñanza en Nutrición y Salud, Cartago, Costa Rica; M. T. Illnait y D. Quiñones, Instituto de Medicina Tropical “Pedro Kouri”, La Habana, Cuba; E. Villancís, J. Reyes, Instituto Nacional de Higiene y Medicina Tropical “L. Izquieta Pérez”, Guayaquil, Ecuador; R. E. Villatoro Ventura, Laboratorio Central “Dr. Max Bloch”, San Salvador, El Salvador; C. Valenzuela, Laboratorio Nacional de Salud, Villa Nueva, Guatemala; C. Morales, Departamento de Laboratorios, Tegucigalpa, Honduras; N. Montes Colima, Instituto de Diagnóstico y Referencia Epidemiológica, Ciudad de México, CDMX, México; J. Ávila, Centro Nacional de Diagnóstico y Referencia, Managua, Nicaragua; R. Ramos y J. Moreno, Laboratorio Central de Referencia en Salud Pública, Panamá, Panamá; M. Martínez Mora, Laboratorio Central de Salud Pública, Asunción, Paraguay; R. Sacsaquispe, Instituto Nacional de Salud, Lima, Perú; R. Ovalles y L. González, Laboratorio Nacional de Salud Pública “Dr. Defilló”, Santo Domingo, República Dominicana; T. Camou, Departamento de Laboratorio de Salud Pública, Montevideo, Uruguay; N. Cuaical, Instituto Nacional de Higiene “Rafael Rangel”, Caracas, Venezuela.

## Financiamiento.

Este trabajo fue posible gracias al generoso aporte del Gobierno de Canadá, a través del Subsidio D-004200–*Combating Antimicrobial Resistance* a la Organización Mundial de la Salud.

## Declaración.

Las opiniones expresadas en este manuscrito son responsabilidad de los autores y no reflejan necesariamente los criterios ni la política de la *RPSP/PAJPH* y/o de la OPS. o la OPS.
